# The relationship between digital literacy and mental health resilience among college students—based on the mediating role of digital learning

**DOI:** 10.3389/fpsyg.2026.1755407

**Published:** 2026-01-28

**Authors:** Xueting Li, Junting Su, Jiaxin Mao, Lin Chen, He Gao, Yongchong Wang, Kuilai Wang, Lei Chen, Hanyu Huang, Qingyu Zhang, Yunxin Ji, Zhenzhen Zhu, Chaolang Fu

**Affiliations:** 1 School of Marxism, Zhejiang University, Hangzhou, China; 2 School of Medicine, Ningbo University, Ningbo, China; 3 Department of Psychiatry and Behavioral Medicine, First Hospital Affiliated to Ningbo University, Ningbo, China; 4 Department of Psychiatry, The Affiliated Kangning Hospital of Ningbo University, Ningbo, China; 5 Department of Psychiatry, Haishu District People’s Hospital, Ningbo, China

**Keywords:** college student, digital learning ability, digital learning engagement, digital literacy, mental health resilience

## Abstract

**Background:**

In the context of educational digital transformation, digital literacy is increasingly recognized as a key psychological resource beyond technical skills. However, the specific mechanisms and group differences through which it influences college students’ psychological resilience remain underexplored. This study examines how digital literacy affects resilience through the mediating roles of digital learning engagement and digital learning ability.

**Methods:**

A survey of 1,256 students at Ningbo University was conducted using convenience sampling. Data were collected via a novel, self-developed Digital Literacy Scale, alongside established instruments for digital learning engagement, digital learning ability, and psychological resilience. Multiple regression and Bootstrap-mediated effect tests, performed in Stata 16, were utilized to rigorously analyze the hypothesized pathways.

**Results:**

Results revealed three key findings: (1) Digital literacy significantly and positively predicted college students’ psychological resilience (*β* = 0.063, *p* < 0.01). (2) Digital learning engagement and digital learning ability collectively demonstrated a significant partial dual-mediating role between digital literacy and psychological resilience. (3) Heterogeneity analyses revealed a differential pattern across subgroups, with stronger positive effects observed among female students and those from rural backgrounds, while an inverse association emerged among students with lower baseline psychological resilience.

**Conclusion:**

This study confirms that digital literacy directly and indirectly has a positive effect on college students’ psychological resilience, through the mediation of digital learning engagement and digital learning ability. These findings support a validated dual-path model, underscoring the practical value of integrating targeted digital literacy training into educational programs aimed at building student resilience.

## 1 Introduction

Under the dual processes of globalization and digitalization, higher education students face increasingly complex psychosocial challenges, placing significant demands on their mental health resilience (Jiang, 2025; Türk et al., 2025). Concurrently, digital literacy is recognized as a critical resource for navigating this environment (Marangell and Randall, 2025).

Although a positive correlation between digital literacy and psychological resilience has been noted (Green and Sheffield, 2022), critical gaps persist regarding its specific mechanisms. First, the pathway is underspecified. Digital engagement may offer support or introduce stressors (Herbert and Manjula, 2022), suggesting complex mediation. Second, studies seldom examine how distinct dimensions of digital literacy differentially impact outcomes. Third, despite global disparities in digital access and training (Siswanto et al., 2022; Sk and Sain, 2024), cross-cultural empirical research on this relationship remains scarce (Pillay, 2023).

**Figure 1:**
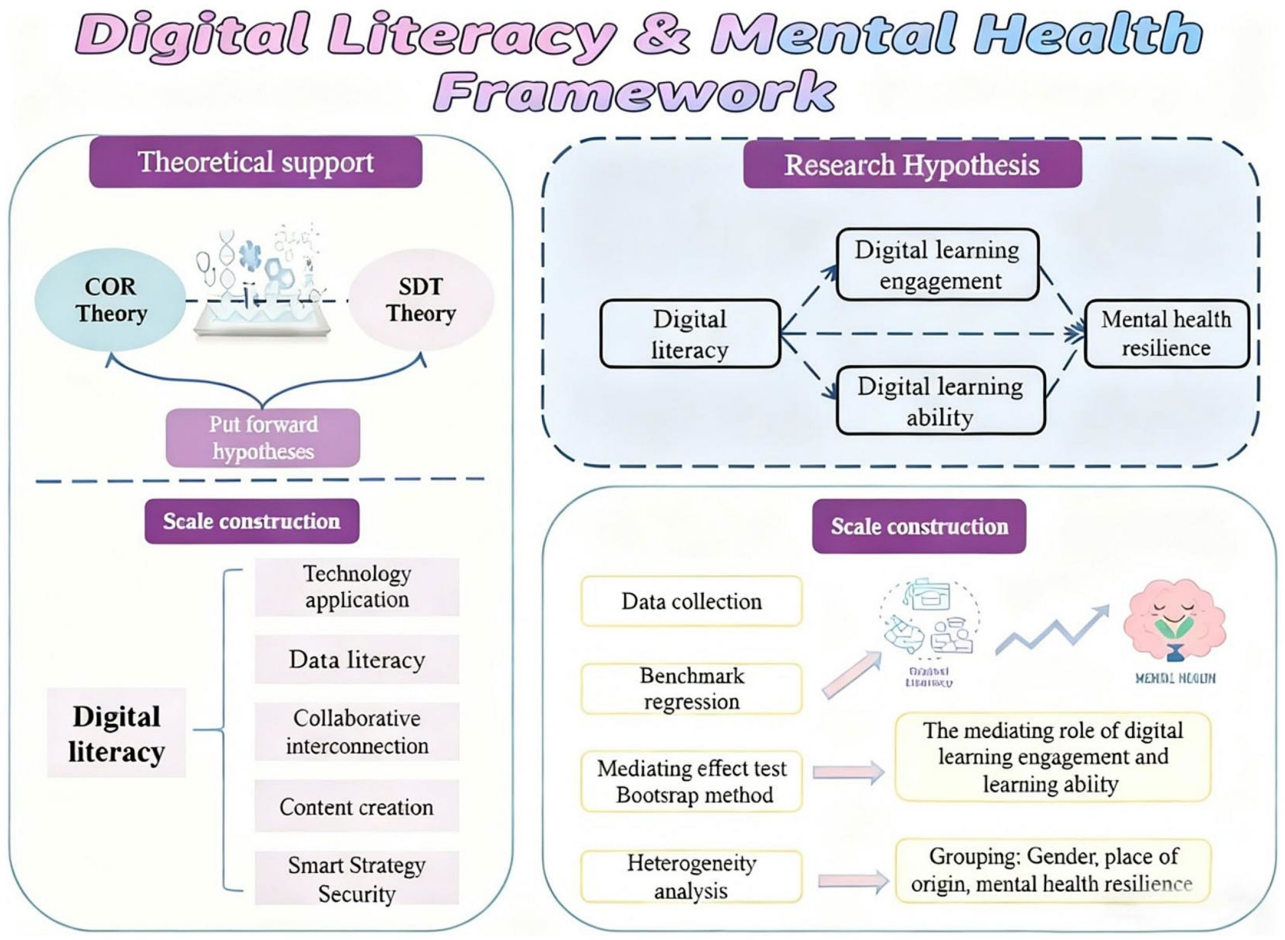
Research framework.

To address these gaps, this study integrates resource conservation theory and self-determination theory to propose and test a sequential mediation model. We investigate whether digital learning engagement and digital competence serve as mediating factors that explain how digital literacy influences mental health resilience among university students, while also examining heterogeneity across cultural contexts. The study aims to: (1) test the direct and indirect effects of digital literacy on resilience; (2) clarify the mediating roles of digital learning engagement and digital competence; and (3) compare these pathways across culturally distinct student groups. This provides targeted empirical evidence for developing culturally adaptive strategies to enhance student wellbeing in digitalized higher education. The specific research framework is shown in Figure 1.

## 2 Literature review and research hypotheses

This paper takes the Resource Conservation Theory (COR) and Self-determination Theory (SDT) as an integrated theoretical framework to construct a theoretical model of the impact of digital literacy on the mental health resilience of college students. The COR theory explains the dynamic process of individual psychological resource gains and losses in the digital environment, while the SDT theory clarifies how these resource states ultimately shape an individual’s motivation and psychological adaptation outcomes by influencing the satisfaction of basic psychological needs. The combination of the two provides a complete “resource—demand—result” logical chain for this study, which is used to systematically deduce the research hypotheses.

In the digital age, the mental health resilience of college students is conceptualized as a dynamic ability to maintain or restore the balance of psychological resources under pressure. The Resource Conservation Theory (COR) states that individuals will strive to acquire, retain and construct resources they consider valuable, such as time, energy, personal traits and social support, in order to prevent the stress caused by resource depletion (Hobfoll, 1989). When under pressure, the depletion of resources will directly threaten physical and mental health. The Self-determination Theory (SDT) further points out that an individual’s mental health and optimal functional performance depend on the degree to which their three basic psychological needs of autonomy, competence and belonging are satisfied (Ryan and Deci, 2000). Environmental resources (as described by COR) can only be effectively transformed into positive psychological outcomes when they are perceived by individuals and used to meet these basic psychological needs. Based on this, from the perspective of COR, digital literacy, as a key personal resource, can help college students acquire and protect other resources more effectively in the digital environment, thereby reducing unnecessary resource waste. More importantly, SDT points out that the effective management of such resources, by empowering individuals to experience greater autonomy, competence and a sense of belonging in core tasks such as digital learning, thereby meeting their basic psychological needs. This state of demand satisfaction is precisely the core manifestation of mental health resilience, enabling individuals to better cope with adversity. Conversely, the lack of digital literacy will lead to the continuous depletion of resources and hinder the satisfaction of demands, intensifying psychological vulnerability.

### 2.1 The impact of digital literacy on mental health

From the perspective of COR theory, digital literacy is a valuable personal resource. The direct impact of digital literacy on the mental health resilience of college students presents a complex duality, serving both as an important enabling resource and a determinant of an individual’s ability to avoid digital risks. According to the resource conservation theory, digital literacy is a type of personal resource. Its existence helps individuals acquire, protect, and utilize other key resources to cope with pressure (Kelly et al., 2022). Specifically, students with high digital literacy can efficiently obtain and evaluate health information, which indicates that they have successfully acquired key information resources and reduced the unnecessary consumption of psychological resources caused by anxiety resulting from information uncertainty. High digital literacy endows college students with the advantage of efficiently obtaining and evaluating health information. For example, students can quickly identify false or panic-inducing information online and accurately locate professional psychological counseling resources and relaxation techniques, significantly reducing anxiety caused by information uncertainty. This proactive ability to seek and screen resources directly reduces the psychological resources depleted by stress, enabling individuals to deal with unexpected events or daily stressors with greater calm and adaptability, demonstrating higher resilience.

Furthermore, digital communication and collaboration skills, a key aspect of digital literacy, help college students build and maintain high-quality social support networks in the digital space. From the perspective of COR theory, a social support network is a crucial social resource, and digital communication skills are the tools for obtaining and maintaining this resource, thereby constructing a resource gain spiral. When facing academic setbacks or emotional distress, individuals who are proficient in using social platforms and instant messaging tools can express their emotions more effectively, seek help and receive perceived social support (Tek and Özsari, 2025). This ability to efficiently utilize digital tools to maintain social connections serves as an important external protective factor that enhances psychological resilience. From the perspective of SDT, successfully applying digital tools to solve problems can directly enhance an individual’s sense of competence. And obtaining support through digital connection fulfills the need for a sense of belonging. The satisfaction of these two basic psychological needs is the internal driving force of psychological resilience. The enhancement of digital self-efficacy stems not only from the experience of successfully mastering digital tools but also directly generalizes to confidence and a sense of control when facing other challenges. High self-efficacy is a core element of mental health resilience (Pellas, 2025).

Conversely, a lack of digital literacy poses a direct threat and source of depletion to psychological resources. According to COR’s “primacy of resource loss” principle, the impact of resource loss is more severe than that of resource acquisition. College students with low digital literacy are more likely to experience social and learning exclusion due to the digital divide. This persistent sense of disadvantage and relative deprivation can easily lead to negative emotions such as low self-esteem, learned helplessness and depression. Prolonged exposure to these emotions can erode an individual’s psychological resilience foundation (Lyu and Luo, 2024). A more direct risk is that the lack of digital security literacy makes individuals vulnerable to cyberbullying (Ning et al., 2024), privacy leaks and digital fraud. These negative events are not only major traumatic stressors, but also directly undermine individuals’ sense of security and self-worth, greatly depleting psychological resources and having a serious impact on the mental health vulnerability of college students (Hu and Meng, 2023). Therefore, the direct role of digital literacy is reflected in its dual influence on an individual’s ability to cope with adversity through resource accumulation and risk avoidance. Based on this, this paper proposes H1.

*H1*: Digital literacy has a positive effect on the mental health resilience of college students.

### 2.2 The impact of digital learning on mental health resilience

Digital learning is not merely the application of technology; it is a process of interaction between individuals and the digital environment. Its impact on mental health resilience depends primarily on two dimensions: the learner’s behavioral commitment and intrinsic capabilities.

Digital learning engagement refers to the degree of effort, attention and participation that college students exert in terms of cognition, emotion and behavior in a digital learning environment. The quality and level of this engagement are directly related to the learning experience and psychological state. According to SDT, a high level of input is the result and external manifestation of the satisfaction of basic psychological needs. According to self-determination theory, high-quality digital learning engagement is a key approach to meeting an individual’s basic psychological needs (Gameil and Al-Abdullatif, 2023). Specifically, active behavioral engagement enables individuals to fulfill their need for a sense of belonging in community interaction. Deep cognitive input enables individuals to meet their need for competence in the process of acquiring knowledge. The autonomous choice of the learning process and methods meets the demand for autonomy. When college students demonstrate high behavioral engagement in online discussions and collaborative projects, they build learning communities through active interactions, fulfilling their need for a sense of belonging. This kind of positive social connection can provide emotional support and resource mutual assistance, which is an important protective factor for psychological resilience and can effectively buffer the sense of loneliness brought about by academic pressure or remote learning (Aryani and Rosyid, 2025). Meanwhile, deep cognitive input, such as critically thinking about online materials and integrating new knowledge with existing knowledge structures, enhances pleasure and competence in knowledge acquisition (Fang et al., 2025). This inner sense of satisfaction constitutes a positive learning experience, reduces frustration during the learning process, and thereby cultivates adaptive psychological resilience to actively deal with challenges. On the contrary, low-quality or imbalanced digital investment, from the perspective of COR theory, is a kind of “resource investment failure” and “resource depletion trap”—individuals invest time and energy resources, but do not obtain the corresponding learning resources or psychological needs to be met, and instead lead to damage in other key resource areas such as studies and social interaction. However, low-quality or overly imbalanced digital engagement, particularly when it becomes digital addiction or passive avoidance, can significantly erode psychological resilience. Digital addiction manifests as a compulsive dependence and uncontrolled use of learning platforms or non-learning digital activities, a typical behavior of resource depletion. This behavior occupies valuable psychological and time resources, seriously interfering with normal study, sleep, and social activities, leading to a decline in academic performance, social isolation, and reduced sleep quality (Dong, 2025). Persistent academic failure and physical imbalance can trigger intense feelings of guilt and anxiety, depleting an individual’s psychological energy to cope with stress, thereby posing a serious vulnerability to psychological resilience.

Digital learning ability refers to an individual’s capacity to effectively utilize tools, manage information, and regulate their own behavior in a digital learning environment. From the perspective of COR theory, digital learning ability is a crucial “resource management strategy,” which determines whether an individual can efficiently transform potential resources in the environment into resources available to the individual and minimize resource consumption in the process. It determines whether an individual can efficiently transform environmental resources into their own advantages (Consoli et al., 2025). High digital learning ability, especially information processing and critical literacy, can help college students effectively optimize their cognitive processes when dealing with massive amounts of digital information. According to cognitive load theory, learners lacking these abilities will generate excessive extraneous cognitive load when confronted with complex multimedia information, encroaching upon the intrinsic cognitive load used for deep learning (Tan et al., 2024). Students with strong digital learning abilities can quickly identify core information, filter out redundant content, and use digital tools for structured organization, reducing external load and alleviating cognitive fatigue and learning anxiety caused by information overload. This effective control over the learning process enhances their sense of self-efficacy in dealing with complex learning tasks, thereby improving their resilience in the face of challenges. The high autonomy of the digital learning environment makes digital self-regulating learning ability a key factor influencing mental health. This ability includes setting clear digital learning goals, choosing appropriate digital learning strategies, and most importantly, time management and emotional monitoring. College students with high self-regulating learning abilities can effectively utilize digital calendars, reminder tools, etc., to allocate their time, and can actively seek online resources for emotional regulation or adjustment of learning strategies when they encounter setbacks in their studies. From the perspective of COR, this successfully avoids the excessive consumption of cognitive resources. From the perspective of SDT, this proficient control over the learning process directly enhances students’ sense of competence. This ability to proactively respond and manage resources is an important manifestation of mental health resilience (Langseth et al., 2023). Conversely, individuals with insufficient self-regulating learning ability are prone to learned helplessness and procrastination, leading to academic failure and accumulated psychological stress, thereby weakening their ability to resist adversity. Therefore, digital learning ability is essentially the core operational resource for college students to maintain mental health and build positive coping mechanisms in the digital age. Based on this, this paper proposes the following hypotheses:

*H2a*: Digital learning engagement has a positive effect on the mental health resilience of college students.

*H2b*: Digital learning ability has a positive effect on the mental health resilience of college students.

### 2.3 The indirect impact of digital literacy on mental health

The promoting effect of digital literacy on mental health resilience is first reflected in optimizing the intermediary path of college students’ engagement in digital learning. In the digital age of information explosion, students with high digital literacy can navigate and process information more efficiently (Yeo et al., 2024), which enables them to reduce cognitive load and search anxiety during the learning process. This mechanism can be strongly supported by self-determination theory. SDT holds that an individual’s autonomy, competence, and sense of belonging to the learning environment are the keys to stimulating intrinsic motivation and enhancing engagement (Wang and Liu, 2008). High digital literacy provides students with the tools to meet these three core needs. First, students who have mastered digital skills can independently choose the most suitable digital resources and tools, such as online collaboration platforms and data visualization software, to effectively manage the learning process, thereby gaining a stronger sense of academic self-efficacy. This sense of efficacy is the direct driving force for cognitive input. Research shows that self-efficacy is significantly positively correlated with psychological resilience (Brosamle et al., 2025). Furthermore, high digital literacy enables students to actively and effectively participate in online learning communities and discussions, gaining positive social support (Benli and Kara, 2025). This sense of social connection and mutual assistance helps to alleviate feelings of isolation and stress, and enhances emotional engagement in learning, such as enthusiasm and pleasure for the learning content. The positive emotions brought about by high emotional investment are the cornerstone of psychological resilience and can help individuals better recover from adversity (Wu et al., 2025). Therefore, digital literacy enhances college students’ sense of effective control, achievement, and positive social experience in a digital learning environment. These positive psychological experiences are transformed into more stable and adaptive mental health resilience through digital learning engagement, effectively buffering the negative impact of academic pressure on mental health.

The second key indirect influence mechanism lies in the improvement of digital capabilities, which constitute the psychological defense line for college students to deal with risks and protect themselves in the digital environment. The cultivation of digital literacy, such as critical assessment and digital security awareness, directly translates into stronger digital citizenship and digital security capabilities. This process can be explained by the resource conservation theory. The COR theory states that the more resources an individual possesses, the more effectively they can withstand stress and trauma and maintain or increase their resource reserves (Liu et al., 2025). Based on this, digital literacy and the resulting digital capabilities are a valuable personal resource. In addition, students with high digital security capabilities can effectively identify risks such as cyberbullying, fraud, and privacy leaks. This preventive risk awareness and operational ability reduce the probability of being harmed by online negative experiences (Tan et al., 2024). The reduction in risk exposure directly translates to a decrease in external digital stressors, indirectly maintaining psychological balance and resilience. Finally, digital literacy can enhance critical thinking and cognitive resilience. Digital literacy emphasizes the ability to critically evaluate online information, that is, the ability to distinguish false information from accurate content (Tang et al., 2025). This ability enhances the cognitive flexibility and digital mental resilience of college students, enabling them to remain calm and rational in analysis and judgment when confronted with uncertain and negative online information, and to avoid falling into negative emotional distress caused by digital anxiety or social comparison (Zhang, 2025). Research shows that the attitude, technology and social dimensions in digital literacy are positively correlated with an individual’s psychological resilience (Tek and Özsari, 2025). In short, digital literacy enhances college students’ sense of control and adaptability to the digital environment by improving their risk-avoidance ability and critical cognitive defense mechanism. Based on this, this paper proposes the following hypotheses.

*H3a*: Digital literacy has a positive effect on the psychological resilience of college students by increasing their engagement in digital learning.

*H3b*: Digital literacy has a positive effect on the psychological resilience of college students by improving their digital learning abilities.

## 3 Research design

### 3.1 Sampling design

#### 3.1.1 Sampling subjects

This study employed convenience sampling to conduct an online questionnaire survey among students from Ningbo University. A total of 1,600 questionnaires were collected. After strict screening, 1,256 valid samples were obtained, yielding a response rate of 78.5%. Participants ranged in age from 17 to 24 years (*M* = 20.3, SD = 1.7 years). The sample comprised 671 males (53.4%) and 585 females (46.6%). The sample included students with varied backgrounds: 211 students with left-behind experiences (16.79%), 551 only children (43.85%), and 489 students from rural areas (38.92%). Regarding learning experiences, 869 students (69.16%) had student leadership experience, and 10 students (0.80%) had experienced school bullying. This study has been approved by the university ethics Review committee. The university ethics review committee approved the study, and all participants provided informed consent after receiving a full explanation of the research purpose. The researchers committed to maintaining the confidentiality of personal information and data.

#### 3.1.2 Data quality control

To ensure the quality of research data, this study adopted the QuestionStar online platform for questionnaire distribution and collection. During the questionnaire design stage, selective questions were specially set up, including three core screening criteria: “Whether one has a full-time college student status,” “whether one has basic digital cognition,” and “whether one understands the basic concepts of digital learning,” to accurately identify the target group and effectively exclude respondents who do not meet the conditions, thereby ensuring the representativeness of the research sample and the validity of the data. To reduce random errors, a uniform standard is adopted for the processing of all open-ended questions. For questionnaires with incomplete responses or insufficient detail, the system prompted respondents to provide additional information. If the quality requirements were unmet after the second attempt, the questionnaire was deemed invalid and excluded from the analysis. Additionally, the QuestionStar platform was configured with control measures to prevent malicious responses and enhance data quality: Each registered ID could only submit the questionnaire once, and a minimum response time of 3 min was required to discourage hasty or casual responses. Furthermore, only college student IDs from the designated sampling regions could be used for submission, thereby ensuring the accuracy and reliability of the data source.

### 3.2 Scale design

#### 3.2.1 Mental health resilience

The Mental Health Resilience (MHR) was measured using a scale originally developed by Connor and Davidson, and subsequently revised by other scholars (Sharif-Nia et al., 2024; Ramzi and Besharat, 2010; Hang et al., 2024). This instrument comprises 25 items, rated on a 5-point Likert scale ranging from 1 (strongly disagree) to 5 (strongly agree). It assesses three core dimensions: strength, resilience, and optimism (Hobbs et al., 2025). A lower total score indicates a lower degree of mental health resilience. The total score for the scale is computed by summing the scores of all items. In the current study, the Cronbach’s alpha coefficient for this questionnaire was 0.98, indicating excellent internal consistency.

#### 3.2.2 Digital literacy (DIGS)

To comprehensively and accurately assess college students’ digital literacy levels, a novel Digital Literacy Scale (DIGS) was developed for this study. Its content was enriched by integrating elements from UNESCO’s Global Digital Literacy framework and the EU’s Dig Comp 2.0. This integration helped to expand the constituent elements and provide thorough explanations. Particular attention was paid to addressing the gap in existing tools regarding the assessment of college students’ digital technology application levels. Furthermore, based on the conceptualization of digital literacy, the scale incorporated questions related to digital knowledge and digital moral ethics, enhancing its adaptability for Chinese college students. The final digital literacy framework consists of five dimensions: technology application, data literacy, collaborative interconnection, content creation and construction, and intelligent security, which are further delineated into 13 constituent elements. Table 1 provides detailed descriptions and explanations of these elements, vividly reflecting the content used to measure college students’ digital literacy.

**Table 1:** Design of digital literacy scale.

Dimension	Components	Description
Technical application	Digital knowledge	Understand principles/scopes of digital tools; know digital regulations/policies.
	Digital skills	Identify/apply hardware functions; grasp info/data in software tools.
Data literacy	Information acquisition	Search/access data; update search strategies.
	Information evaluation	Critically assess credibility of data/info sources.
	Information management	Organize and store data/info/digital content.
Collaborative connection	Digital communication and collaboration	Interact/share/collaborate via digital technologies.
	Digital Identity Management	Follow norms for accounts/passwords; manage digital reputation/identity.
Content creation	Content creation	Edit digital content in various forms to express ideas.
	Content integration	Integrate/refine content to create new works.
	Copyright and licensing	Understand rules of copyright/licenses for digital info/content.
Wisdom, strategy and security	Problem-solving	Resolve digital environment issues; select tools; improve digital capabilities.
	Digital security	Protect privacy/data; avoid psychological risks; maintain academic integrity.
	Digital ethics and morality	Abide by moral/legal norms; spread info legally/appropriately.

To address the essential need for robust psychometric evidence, the scale’s properties were rigorously evaluated using a sample-splitting approach.

##### 3.2.2.1 Exploratory factor analysis

In the randomly selected half of the samples, the results of exploratory factor analysis using principal factorization and Promax rotation method showed that the scale presented a clear five-factor structure, which was in complete agreement with the theoretical model. As shown in Table 2, the Kaiser-Meyer-Olkin sampling fitness measure is 0.94, and the Bartlett sphericity test results are significant, indicating that the data are suitable for factor analysis. The loading of all items on their corresponding factors ranges from 0.62 to 0.89, and there is no obvious cross-factor loading, indicating that the factor structure is clear and interpretable.

**Table 2:** Summary of exploratory factor analysis (EFA) results.

Dimension	Number of Items	KMO Value	Bartlett’s test (χ^2^)	Factor loading range	Eigenvalue (>1)	Cumulative variance explained (%)
Technical application	4	0.94	8560.32*	0.75–0.89	8.21	63.15
Data literacy	3			0.72–0.86	1.87	77.53
Collaborative connection	3			0.68–0.82	1.42	88.48
Content creation	3			0.62–0.79	1.15	97.35
Wisdom and security	3			0.65–0.84	1.03	100

*Indicates significance at the 10% level.

##### 3.2.2.2 Confirmatory factor analysis and validity assessment

According to the confirmatory factor analysis results in Table 3, the five-factor model data fits well on the other half of the samples, with specific indicators being $\chi^{2}$ /df = 2.18, CFI = 0.96, TLI = 0.95, RMSEA = 0.051, and SRMR = 0.039. All standardized factor loadings were significant and ranged from 0.68 to 0.92. The average variance extraction of each latent variable ranged from 0.53 to 0.71, all exceeding the standard threshold of 0.50. The combined reliability ranged from 0.87 to 0.94, supporting the aggregated validity of the scale. Discriminant validity analysis indicates that the square roots of the average variance extraction of each latent variable are all greater than their correlation coefficients with other latent variables. In terms of reliability, the Cronbach’s α coefficients of each dimension range from 0.86 to 0.93, with a total expression reaching 0.98. Although a higher total scale coefficient may reflect some overlap among the items, this is closely related to the multi-dimensional structure of the scale and the homogeneity of the samples. Importantly, the clear factor structure, ideal fit indicators and satisfactory validity and reliability evidence provided by confirmatory factor analysis jointly support that this scale has qualified psychometric attributes.

**Table 3:** Confirmatory factor analysis (CFA) results and psychometric properties.

Test category	Index	Result	Criterion	Conclusion
Overall model fit	χ^2^/df	2.18	<3.0	Excellent
	CFI	0.96	>0.90	Excellent
	TLI	0.95	>0.90	Excellent
	RMSEA	0.051	<0.08	Good
	RMSEA 90% CI	0.047–0.055		
	SRMR	0.039	<0.05	Excellent
Convergent validity	AVE range	0.53–0.71	>0.50	Satisfactory
	Factor loading range	0.68–0.92*	>0.50	Satisfactory
Discriminant validity	√AVE > Inter-construct correlations	Yes	√AVE > r	Satisfactory
Composite reliability	CR range	0.87–0.94	>0.70	Satisfactory
Internal consistency	Cronbach’s α (overall)	0.98	>0.70	Excellent
	Cronbach’s α (subscales)	0.86–0.93	>0.70	Excellent

*Indicates significance at the 10% level.

#### 3.2.3 Mediating variables

The study utilized existing assessment tools for Digital Learning Engagement (DIGTR) as a foundational reference for developing its own online learning engagement scale. Based on the conceptual definition of online learning engagement, the scale was structured around four dimensions: behavioral, cognitive, emotional, and social interaction engagement. The specific items were carefully compiled and revised to align with the unique characteristics of college students (Aryani and Rosyid, 2025). The Cronbach’s alpha coefficient for this questionnaire was 0.98, indicating high internal consistency.

Similarly, Digital Learning Ability (DIGLE) was evaluated using a two-dimensional framework focusing on learning outcomes and behavioral changes, adapted from existing measurements (Lee and Chang, 2025). This scale was also tailored for college students and demonstrated excellent reliability. In the present study, the Cronbach’s alpha coefficient for the Digital Learning Ability questionnaire was 0.98.

#### 3.2.4 Control variables

To more accurately estimate the net effect of digital literacy on college students’ mental health resilience, this study controlled for a series of demographic and key experience variables identified in existing theories and literature (Parveen et al., 2025; Younis, 2025). These variables, which could potentially confound the relationship between digital literacy and psychological resilience, included: (1) Basic demographic characteristics: Gender (GENDER), Age (AGE), Education (EDU), and place of origin (URBAN); (2) School Experience and Role (SCE): Whether the student had experience as a student leader; (3) Potential traumatic experiences: Whether the student had a history of being left behind (LBE) or had experienced school bullying (SCBE). By incorporating these control variables into the model, the interference caused by these factors could be effectively mitigated, thereby allowing for a clearer delineation of the independent correlation between digital literacy and psychological resilience.

### 3.3 Data processing

This study utilized an online questionnaire for data collection. Researchers ensured participant anonymity and explained the research purpose and related procedures to all participants before they completed the questionnaire. Descriptive statistics, difference tests, and correlation analyses were performed on the sample data using Stata 16. Subsequently, a regression model was applied, and the Bootstrap analysis method, involving 5,000 resamples, was used to test the significance of the mediating effects on psychological resilience.

## 4 Empirical results

### 4.1 Common method bias test

To assess common method bias in the sample, Harman’s single-factor test was conducted. The results indicated the presence of four factors with eigenvalues greater than 1. The first factor accounted for 34.46% of the variance, which is below the 40% critical threshold. This suggests that common method bias is not a significant concern in this study.

### 4.2 Descriptive statistics

This study used a five-point Likert scale to measure the items, with responses ranging from 1 (strongly disagree) to 5 (strongly agree). The descriptive statistics for each key variable are presented in Table 4. The overall level of mental health resilience (MHR) among college students was above average (*M* = 2.721, SD = 0.289), with scores ranging from 1.800 to 3.760. The mean values for the core independent variable digital literacy (DIGS), digital learning engagement (DIGTR), and digital learning ability (DIGLE) were 3.209, 3.063, and 2.810, respectively. All three variables had mean values higher than the theoretical median, indicating that the college students surveyed generally possess a good level of digital literacy and exhibit a relatively positive state of digital learning. Meanwhile, the standard deviations for each variable suggest a certain degree of discreteness in the data, which is suitable for further analysis.

**Table 4:** Descriptive statistical results of variables.

Variable	Obs	Mean	SD	Min	Max
MHR	1,256	2.721	0.289	1.800	3.760
DIGS	1,256	3.209	0.473	2.154	4.231
DIGTR	1,256	3.063	0.335	1.625	4.250
DIGLE	1,256	2.810	0.366	1.455	3.909
Gender	1,256	0.533	0.499	0.000	1.000
AGE	1,256	2.631	0.977	1.000	4.000
URBAN	1,256	0.615	0.487	0.000	1.000
EDU	1,256	3.659	1.041	1.000	5.000
SCE	1,256	0.692	0.462	0.000	1.000
LBE	1,256	0.168	0.374	0.000	1.000
SCBE	1,256	0.008	0.089	0.000	1.000

Regarding demographic variables, the sample showed a relatively balanced ratio of men to women, and the average age corresponded to the mid-university stage. Statistics on students’ place of origin indicated a slightly higher proportion of urban students compared to rural students. The educational attainment of parents was relatively high, with an average of junior college or higher. In terms of school experiences, the majority of students reported positive campus experiences, while 16.8% had experiences of being left behind. The incidence of school bullying experiences was very low, at only 0.8%. All variable values fell within a reasonable range, confirming the good data quality for subsequent statistical analysis.

### 4.3 Correlation analysis

Analysis of the Pearson correlation coefficients, presented in Table 5, revealed several significant relationships. First, mental health resilience showed a significant positive correlation with digital literacy, digital learning engagement, and digital learning ability. The strongest correlation was observed with digital learning engagement (*r* = 0.268), followed by digital literacy (*r* = 0.183), and then digital learning ability (*r* = 0.107). This finding initially supports the research hypotheses, indicating a close relationship between digital literacy, its application in the learning process, and the development of psychological resilience among college students. In terms of controlling variables, mental health resilience was significantly negatively correlated with age and place of origin, Conversely, it was significantly positively correlated with experiences of being left behind and school bullying. Notably, a strong negative correlation (*r* = −0.378) was found between school experience and mental health resilience. However, gender and the educational attainment of parents did not show a significant correlation with mental health resilience.

**Table 5:** Results of the person correlation coefficient matrix.

Variable	MHR	DIGS	DIGTR	DIGLE	GENDER	AGE	URBAN	EDU	SCE	LBE	SCBE
MHR	1										
DIGS	0.183***	1									
DIGTR	0.268***	0.196***	1								
DIGLE	0.107***	0.091***	0.281***	1							
GENDER	−0.016	0.042	−0.026	−0.102***	1						
AGE	−0.122***	−0.080***	−0.160***	0.009	−0.002	1					
URBAN	−0.104***	0.022	0.099***	0.027	0.022	0.018	1				
EDU	0.026	−0.004	−0.022	−0.036	−0.004	0.048*	−0.004	1			
SCE	−0.378***	−0.207***	−0.232***	−0.132***	0.043	0.168***	−0.014	−0.076***	1		
LBE	0.057**	0.117***	0.048*	0.049*	0.100***	0.024	0.145***	−0.025	−0.069**	1	
SCBE	0.125***	0.058**	0.065**	−0.094***	0.084***	−0.021	0.034	0.012	−0.095***	0.056**	1

***Indicates significance at the 1% level, **Indicates significance at the 5% level, and *Indicates significance at the 10% level.

The correlation coefficients among the selected explanatory variables were all less than 0.8, indicating no significant multicollinearity. Multicollinearity tests were conducted on the variables. To further confirm this, Variance Inflation Factor (VIF) values were calculated for each explanatory variable. As shown in Table 6, the VIF values and tolerance for all variables met the accepted standards, thus confirming the absence of multicollinearity issues.

**Table 6:** Values of variable VIF.

Variable	DIGTR	SCE	DIGLE	DIGS	AGE	LBE	SCBE	URBAN	AGE	EDU
VIF	1.20	1.14	1.13	1.09	1.06	1.05	1.04	1.03	1.03	1.01
1/VIF	0.84	0.88	0.89	0.92	0.95	0.95	0.96	0.97	0.97	0.99

### 4.4 Benchmark regression

This study investigated the impact of digital literacy on the mental health resilience of college students using a regression model, with the benchmark regression results presented in Table 7. Model 1 only included the core independent variable, digital literacy, while Model 2 further incorporated all control variables. Firstly, digital literacy showed a significant positive predictive effect on the mental health resilience of college students. In Model 1 without control variables, the regression coefficient for digital literacy was 0.112 and was significant at the 0.1% level (*p* < 0.001). After including all control variables in Model 2, the coefficient for digital literacy decreased to 0.063 (*p* < 0.01) but remained statistically significant. This indicates that, even when controlling for other influencing factors, a one-unit increase in digital literacy among college students is associated with a 0.063-unit increase in their mental health resilience, thereby confirming Hypothesis H1.

**Table 7:** Benchmark regression results.

Variables	(1)	(2)
	MHR	MHR
Digs	0.112*** (6.1284)	0.0630*** (3.6046)
GENDER		−0.00818 (−0.5555)
AGE		−0.0161** (−2.1018)
URBAN		−0.0702*** (−4.6472)
EDU		0.000645 (0.0962)
SCE		−0.211*** (−12.1204)
LBE		0.0287 (1.3326)
SCBE		0.288** (2.4228)
Constant	2.361*** (40.5060)	2.746*** (39.0671)
Observations	1,256	1,256
R-squared	0.0336	0.1786

***Indicates significance at the 1% level, **indicates significance at the 5% level, and * indicates significance at the 10% level. The values in parentheses are *t*-values.

Secondly, significant differences were observed in the impact of various control variables on mental health resilience. School experience (SCE) exhibited the strongest negative predictive power (*β* = −0.211). A significant negative correlation was also found between mental health resilience and place of origin (URBAN), with urban students showing lower resilience than rural students. Older age was associated with lower levels of psychological resilience (AGE, *β* = −0.0161). Interestingly, experiences of school bullying (SCBE) were significantly positively correlated with psychological resilience (*β* = 0.288). Although this finding is contrary to intuition, it can be explained from the dual perspectives of post-traumatic growth theory and resilience development: moderate exposure to adversity may activate an individual’s psychological resource reorganization process, promoting the development of self-efficacy, the ability to seek social support, and emotional regulation strategies through coping with challenges. In other words, some students may develop more adaptive psychological defense mechanisms and coping skills in the process of dealing with bullying experiences, thereby demonstrating stronger psychological resilience in subsequent stressful situations. This conforms to the adaptive development path of “adversity exposure—activation of psychological resources.” In contrast, factors such as gender, parents’ educational attainment (EDU), and left-behind experience (LBE) did not show significant predictive effects. From the perspective of the model’s explanatory power, the R^2^ increased from 0.0336 in Model 1 to 0.1786 in Model 2 after adding control variables. This indicates that the included variables collectively explained 17.86% of the variance in psychological resilience.

### 4.5 Mediating effect test

The results of the mediating effect test, based on stepwise regression analysis in this paper, clearly elucidated the internal mechanism by which digital literacy affects mental health resilience. As shown in Table 8, digital literacy not only directly has a positive effect on mental health resilience but also exerts an indirect influence through two parallel paths: digital learning engagement and digital learning ability.

**Table 8:** Regression results of mediating effects.

Variables	(1)	(2)	(3)	(4)
	Digtr	MHR	Diglearn	MHR
Digs	0.103*** (5.0981)	0.0466*** (2.6616)	0.0552** (2.4745)	0.0602*** (3.4297)
Digtr		0.159*** (5.9640)		
Diglearn				0.0514** (2.2764)
Constant	2.931*** (35.5539)	2.280*** (21.9315)	2.751*** (29.8000)	2.604*** (26.6621)
Controls	Yes	Yes	Yes	Yes
Observations	1,256	1,256	1,256	1,256
R-squared	0.1020	0.2090	0.0475	0.1826

***Indicates significance at the 1% level, **indicates significance at the 5% level, and *indicates significance at the 10% level. The values in parentheses are *t*-values.

In the analysis of Path One, digital literacy demonstrated a significant positive predictive effect on digital learning engagement, with a standardized coefficient reaching 0.103 and being significant at the 0.1% level (*p* < 0.001). This indicates that higher digital literacy levels among college students are associated with deeper participation and sustained investment in the digital learning environment. Subsequently, digital learning engagement significantly predicted mental health resilience, with an impact coefficient of 0.159 (*p* < 0.001), thus confirming Hypothesis H2a. This discovery reveals the transmission mechanism by which digital literacy influences mental health by promoting learning engagement: students with good digital skills can make more effective use of digital learning resources, thereby gaining more sense of achievement and control during the learning process. These positive experiences ultimately translate into enhanced psychological resilience.

In the analysis of Path Two, the study discovered another parallel mediation mechanism. Digital literacy had a significant positive impact on digital learning ability, with a coefficient of 0.0552 (*p* < 0.01), indicating that the improvement of digital literacy directly has a positive effect on the development of students’ learning ability in a digital environment. Digital learning ability, in turn, significantly promoted mental health resilience, with a coefficient of 0.0514 (*p* < 0.01), thereby confirming Hypothesis H2b. This path highlights the significant value of ability construction, where digital literacy helps students build confidence in academic challenges by enhancing their sense of learning efficacy in the digital environment, and this confidence generalizes to the psychological ability to cope with life pressure.

It is important to note that when both mediating variables were included in the model, the direct effect of digital literacy on mental health resilience decreased from the initial 0.112–0.0602, yet remained statistically significant (*p* < 0.001). This statistical feature clearly indicates that both digital learning engagement and digital learning ability play a partial mediating role between digital literacy and mental health resilience, thus confirming Hypotheses H3a and H3b. This means that digital literacy not only indirectly cultivates psychological resilience by enhancing learning engagement and learning ability but also has a positive effect on psychological resilience. This direct effect may stem from other paths not included in the model, such as the information acquisition advantage and expanded social support networks facilitated by digital literacy.

To further verify the mediating effects, a bootstrap test was performed. As presented in Table 9, for the path “DIGS -DIGTR -MHR,” the confidence interval did not contain zero, and the direct effect remained significant, indicating that digital learning engagement partially mediates the relationship between digital literacy and mental health resilience. Similarly, the significant mediating role of digital learning ability was confirmed for the path “DIGS -DIGLE -MHR” by a confidence interval excluding zero.

**Table 9:** Bootstrap test results.

Path	Eq	Coef.	Std. Err.	*z*	P > z	[95% Conf. Interval]	
DIGS-DIGTR-MHR	_bs_1	0.029	0.006	5.160	0.000	0.018	0.040
	_bs_2	0.083	0.019	4.450	0.000	0.047	0.120
DIGS-DIGLE-MHR	_bs_1	0.005	0.002	2.170	0.030	0.000	0.010
	_bs_2	0.107	0.018	5.830	0.000	0.071	0.143

### 4.6 Heterogeneity analysis

This study conducted an in-depth exploration of the heterogeneity of digital literacy’s impact on mental health resilience through group regression. The results, detailed in Table 10, revealed significant differences across groups based on gender, place of origin, and initial levels of psychological resilience, offering crucial empirical evidence for targeted interventions.

**Table 10:** Results of heterogeneity test.

Variables	Group: gender		Group: place of origin		Group: the level of MHR	
	(1) Male	(2) Female	(3) Urban	(4) Rural	(5) Lower MHR level	(6) Higher MHR level
	MHR	MHR	MHR	MHR	MHR	MHR
Digs	0.0476* (1.7632)	0.0791*** (3.5793)	0.0476* (1.7632)	0.0791*** (3.5793)	−0.0916*** (−4.0753)	0.0802*** (5.6369)
Constant	2.795*** (25.9931)	2.688*** (31.1022)	2.795*** (25.9931)	2.688*** (31.1022)	2.678*** (28.9237)	2.650*** (44.6705)
Controls	Yes	Yes	Yes	Yes	Yes	Yes
Observations	670	586	670	586	264	992
R-squared	0.1606	0.2076	0.1606	0.2076	0.1385	0.1760

*Indicates significance at the 10% level, ***Indicates significance at the 1% level.

Firstly, comparing the gender grouping results (models 1 and 2 in Table 10), digital literacy’s promoting effect on psychological resilience was significantly more pronounced among female students (*β* = 0.0791, *p* < 0.001) than among male students (*β* = 0.0476, *p* < 0.05). This difference may stem from distinct patterns in how men and women utilize digital technology and seek social support. Women often leverage digital technology to establish and maintain social connections and gain emotional support through online social networks. The construction of such support networks can amplify the protective effect of digital literacy on psychological resilience. Additionally, women tend to be more proactive in seeking help when facing psychological distress, and digital literacy enables them to access mental health resources and professional support more conveniently, thereby more effectively transforming digital capabilities into psychological capital.

Secondly, the grouping results based on student origin (models 3 and 4 in Table 10), indicated that rural students benefited more from improved digital literacy. The coefficient for the rural student group was 0.0791 (*p* < 0.001), significantly higher than that for the urban student group (*β* = 0.0476, *p* < 0.05). This founding has significant implications for educational equity. Digital technology can provide rural students with an important means to overcome resource disadvantages. For rural students, digital literacy is not merely a skill but a key tool to surmount geographical limitations, access high-quality educational resources, and broaden their horizons. Through digital platforms, they can access learning opportunities and social networks that were previously out of reach. This “leap effect” in resource acquisition can significantly enhance their psychological adaptability. In contrast, urban students often already possess relatively abundant resources, thus the marginal benefit of digital literacy may be smaller for them.

Finally, we divided the samples into high initial resilience groups and low initial resilience groups based on the median of psychological resilience. The results of the group regression are shown in Model (3) and Model (4) in Table 10. For the group with higher initial psychological resilience, digital literacy showed a significant promoting effect (*β* = 0.0802, *p* < 0.001); However, in the group with lower initial resilience, digital literacy showed a significant negative impact (*β* = −0.0916, *p* < 0.001). This divergent result reveals the complexity of the impact of digital literacy.

Individuals with high psychological resilience may possess strong self-regulation and critical thinking abilities, be able to effectively screen and utilize digital resources, avoid negative effects of the Internet, and thereby transform digital literacy into a psychological advantage. The negative associations that emerged in the low-resilience group can be systematically explained by combining the “differential sensitivity” framework and the “stress-vulnerability” model. From the perspective of differential sensitivity, digital literacy, as an environmental resource, does not have a homogeneous impact: highly resilient individuals may exhibit more “opportunity sensitivity,” actively using digital tools for learning, connection, and self-improvement, thereby amplifying its positive effects. Individuals with low resilience may exhibit “fragile sensitivity,” meaning that the same level of digital exposure will make them more sensitive to threats such as social comparison, cyberbullying, and information overload in the digital environment, accelerating the consumption of their already limited cognitive and emotional resources. Furthermore, by further integrating digital stress theory with the stress-vulnerability model, low psychological resilience itself implies a more vulnerable internal baseline state and more limited psychological buffer resources. Although digital literacy grants them more access rights, if they lack corresponding digital coping strategies and external psychological support, their increased digital participation may disproportionately translate into stress experiences, especially the “constant online” stress and social comparison pressure that are widespread in the digital environment. This not only fails to meet their basic psychological needs of autonomy, competence and relationship belonging, but may also induce or intensify the sense of demand frustration, thus forming a negative path between digital literacy and psychological adaptation.

## 5 Conclusion and suggestions

### 5.1 Research conclusion

This study, through an empirical investigation of 1,256 Chinese college students, revealed the intrinsic mechanisms underlying the interaction among digital literacy, digital learning engagement, digital learning ability, and mental health resilience. The core conclusions are as follows: First, digital literacy, recognized not only as a key skill but also as a psychological resource, has a significant positive predictive effect on college students’ psychological resilience. Second, improvements in digital literacy do not directly influence psychological resilience in isolation, but rather foster it indirectly through a dual mediation pathway: by stimulating higher levels of digital learning engagement and subsequently transforming this engagement into effective digital learning capabilities. Finally, this promoting effect exhibits group heterogeneity being more pronounced among female students, those from rural areas, and students with lower initial psychological resilience levels. This highlights the substantial potential of digital empowerment strategies in promoting educational equity and supporting psychologically disadvantaged groups. In conclusion, systematically enhancing students’ digital literacy at the higher education stage is an effective approach to build their psychological “immune system” to cope with the challenges of future society.

### 5.2 Policy recommendations

At present, digital literacy courses in colleges and universities mostly focus on tool operation and have a weak connection with students’ psychological growth goals. Research has confirmed that digital literacy can only be effectively transformed into psychological resilience by promoting high-quality digital learning investment and ability development. Therefore, curriculum reform should go beyond the traditional fundamentals of computer science and build three core modules: “Tool empowerment,” “cognitive regulation,” and “resource seeking.” The tool empowerment module aims to teach students how to use digital tools to enhance their learning efficiency and sense of order, meeting their need for competence. The cognitive regulation module should focus on cultivating students’ critical thinking about online information and their self-monitoring ability regarding digital usage behavior, helping them maintain rationality and concentration in complex information environments and protect their cognitive resources. The resource seeking module should guide students to proactively identify and utilize reliable online mental health resources and social support networks, thereby meeting their need for a sense of belonging and actively building social resources. This curriculum design aims to systematically transform the digital environment from a potential source of stress into a resilient training ground for students to cultivate self-management skills, rational cognitive habits, and proactive help-seeking behaviors.

Research has found that the promoting effect of digital literacy is more significant or complex among female students, rural students, and students with low initial psychological resilience. This provides a clear direction for implementing precise intervention. Educational institutions should establish an early identification mechanism that integrates learning behavior data with psychological screening. By analyzing anonymous data such as learning engagement and resource access patterns, and combining with regular psychological scale screening, student groups with potential risks in digital adaptation or psychological states are dynamically identified. On this basis, provide stratified and categorized intervention resource packages. For instance, focus on offering basic skills bridging courses and equipment support to rural students with lower digital access levels. For students with low psychological resilience, it is necessary to organically integrate emotional management strategies, stress coping skills, and guidance on how to use digital tools for self-care into digital skills training, and to smooth out offline channels for them to obtain professional psychological support. In view of the possible negative effects of digital literacy on students with low resilience found in this study, the intervention plan must adhere to the principle of “simultaneous improvement of literacy and psychological support,” and avoid merely providing technical training to increase their psychological burden.

The cultivation of digital psychological resilience cannot be undertaken by a single course; it requires the formation of a collaborative system within universities. The student affairs department and the psychological counseling center need to be deeply involved in the planning of digital literacy education and provide professional content and channels for mental health support. The Teacher Development Center should train teachers to design digital learning activities in professional course teaching that can promote students’ autonomy, competence and sense of belonging. The information technology department needs to ensure the ease of use and accessibility of digital tools and assist in developing data monitoring tools that protect students’ privacy. Only by establishing a three-in-one collaborative working mechanism of “course teaching—student support—technical services” can the cultivation of digital literacy be comprehensively and throughout the entire process integrated into the student growth ecosystem, and can resilient individuals who can master technology in the digital age and nourish their inner selves be systematically cultivated.

## 6 Discussion

This study elevates the theoretical connotation of digital literacy from the technical operation level to the psychological resource level. Existing studies have shown that digital ability can promote mental health by enhancing a sense of control and self-efficacy (Lorini et al., 2023), and digital literacy also helps alleviate Internet anxiety among college students. This study reveals the continuous mediating role played by digital learning engagement and digital learning ability in it. This discovery is in line with the framework constructed in this study by integrating self-determination theory (SDT) and resource conservation theory (COR): Digital learning input reflects the psychological process of satisfying the needs of autonomy, competence and relationship, while digital learning ability represents the adaptive mechanism for efficient resource management and investment in the academic field (Yan et al., 2025). This means that in the environment of higher education where learning is the core task, digital literacy can more directly affect the psychological adaptation process through its specific application in the academic field.

The findings of group heterogeneity in this study have significant implications. Unlike existing studies suggesting that digital literacy has a more significant impact on men’s technological confidence (Chen et al., 2024), this study found that the promoting effect of digital literacy on women’s psychological resilience is more prominent. This difference may be related to the strategies that women are better at transforming digital technology into social support and emotional resources in specific cultural contexts (Fang et al., 2025). Meanwhile, the significant positive effects of digital literacy on rural students confirm the “digital inclusion” theory, indicating that technology may bring higher marginal benefits to groups with relatively scarce resources.

Particularly crucial is that this study identified the complex influence pattern of digital literacy on students with low initial psychological resilience (*β* = −0.0916, *p* < 0.001). This discovery constitutes an important revision to the “technology neutrality theory,” indicating that the psychological benefits of digital technology may depend on an individual’s existing level of psychological vulnerability. Starting from the “loss spiral” principle of resource conservation theory and the stress-vulnerability model, individuals who originally lack psychological resources may be more vulnerable to the erosion of pressures such as information overload, negative social comparison, and cyberbullying in the broader digital environment exposure brought about by the improvement of digital literacy (Hobfoll, 1989), accelerating the depletion of their psychological resources.

### 6.1 Study limitations

This study significantly contributes to understanding the intricate mechanisms through which digital literacy influences the mental health resilience of college students. Our findings illuminate a dual-mediated pathway involving digital learning engagement and digital learning ability, underscoring the practical value of integrating digital literacy training into educational programs aimed at fostering student resilience. Furthermore, the heterogeneity analyses provide crucial insights into how the effects of digital literacy vary across different demographic groups and initial resilience levels, offering targeted directions for intervention strategies.

Despite its valuable contributions, this study is subject to several limitations that warrant consideration for future research. Firstly, the cross-sectional design employed in this research inherently precludes the establishment of definitive causal relationships. While our models indicate plausible mediating pathways, they primarily reveal associations between variables rather than causal mechanisms. Consequently, future longitudinal investigations are essential to unravel the dynamic interplay and confirm the causal mechanisms linking digital literacy to mental health resilience over time. Secondly, the reliance on convenience sampling, specifically focusing on students from a single institution (Ningbo University), constrains the generalizability of our findings. This sampling strategy may limit the applicability of our results to broader student populations, including those from other regions or with diverse educational backgrounds. Therefore, replication with more diverse and nationally representative samples is warranted to enhance external validity and confirm the robustness of our conclusions.

Thirdly, the exclusive use of self-reported questionnaires, while efficient, carries inherent limitations. Despite Harman’s single-factor test indicating no significant common method bias, the potential for social desirability bias and subjective interpretations cannot be entirely ruled out. A specific and related limitation arises from the fact that digital literacy itself was measured via a digital, self-report survey. This method may introduce a systematic sampling bias, as students with very low digital literacy or limited digital access might have been less likely or able to participate in an online study. Consequently, our sample may under-represent this subpopulation, potentially leading to a restricted range in digital literacy scores and an overestimation of its average level and its positive associations with other constructs. Our findings, therefore, may best generalize to students who are already sufficiently digitally literate to engage with an online survey. Future research could benefit from integrating objective measures or multi-source data, and could employ mixed-mode data collection (e.g., combining online with paper-based or assisted digital methods) to better capture the full spectrum of digital literacy levels and mitigate this form of participation bias.

Finally, the study’s exclusive focus on a specific cultural context in China, while providing valuable region-specific insights, implies that certain findings, particularly concerning heterogeneity effects, may not be directly transferable to diverse cultural settings. Thus, cross-cultural comparative studies are vital to delineate the universality and specificity of these relationships and enrich the global understanding of digital literacy and mental health resilience.

### 6.2 Future outlook

Based on these findings, future research and practice can pursue deeper exploration in both theoretical construction and research methods. At the theoretical level, future research should focus on constructing a theoretical framework that explains the differentiated impacts of digital literacy. Specifically, it should integrate key moderating variables to systematically explain under what conditions and for which specific groups digital literacy is most likely to transform into psychological assets. This theoretical construction requires full consideration of Chinese unique social and cultural background, in-depth exploration of how local factors such as family expectations and collectivist values regulate the relationship between digital literacy and mental health, and a deeper understanding of the negative impact mechanisms of digital literacy, especially the potential risks for psychologically vulnerable groups. In terms of research methods, future efforts should actively promote longitudinal research designs and long-term follow-up investigations to accurately capture the dynamic interaction between digital literacy and psychological resilience. Intensive longitudinal data collection methods such as empirical sampling, can be introduced to precisely reveal the causal relationship between daily fluctuations in digital usage and changes in psychological states. Simultaneously, it is necessary to conduct multi-dimensional and refined measurements of digital literacy and psychological resilience, develop localized scales suitable for Chinese college students, and combine mixed research methods to deeply understand the subjective experiences of college students’ digital life through qualitative interviews, providing a rich explanation for quantitative research.

## Data Availability

The original contributions presented in the study are included in the article/supplementary material, further inquiries can be directed to the corresponding authors.
